# Comparison of double-layer large-diameter and conventional small-diameter plastic stents for preoperative biliary drainage in resectable distal malignant biliary obstruction

**DOI:** 10.1038/s41598-020-70183-y

**Published:** 2020-08-06

**Authors:** Tokuhiro Matsubara, Tsutomu Nishida, Shiro Hayashi, Hiromi Shimakoshi, Yoshito Tomimaru, Kei Takahashi, Dai Nakamatsu, Kengo Matsumoto, Masashi Yamamoto, Masami Inada

**Affiliations:** 1 Department of Gastroenterology, Toyonaka Municipal Hospital, 4-14-1 Shibahara, Toyonaka, Osaka 560-8565 Japan; 2 Department of Gastroenterology and Internal Medicine, Hayashi Clinic, Suita, Japan; 3 Department of Surgery, Toyonaka Municipal Hospital, Toyonaka, Japan; 4 Department of Surgery, Graduate School of Medicine, Osaka University, Suita, Japan

**Keywords:** Bile duct cancer, Pancreatic cancer

## Abstract

The use of a plastic stent (PS) in resectable patients with distal malignant biliary obstruction (DMBO) is uncommon due to the high failure rate of this method. This study evaluated the efficacy and safety of a double-layer, large-diameter PS as a bridge to surgery compared with a conventional PS. This was a single-center retrospective cohort study. In total, 129 consecutive patients with DMBO underwent pancreaticoduodenectomy between January 2011 and March 2018. Fifty-five patients who preoperatively underwent plastic biliary drainage were enrolled. The patients were divided into two groups based on stent diameter: a large-diameter plastic stent (LPS) group and a small-diameter plastic stent (SPS) group. The primary endpoint was the stent patency period, and the secondary endpoint was the medical cost. Thirty-six patients received SPSs; 19 patients received LPSs. The patency rate until surgery was significantly higher in the LPS group than in the SPS group (89.5% vs. 41.7%, P = 0.0006). Multivariate analysis revealed that LPS use was significantly associated with sufficient stent patency. The total cost of LPS use was significantly lower than that of SPS use. LPSs had longer patency and reduced medical costs than SPSs. LPSs may be suitable for patients with DMBO who are scheduled to undergo surgery.

## Introduction

Distal malignant biliary obstruction (DMBO) is a frequent complication caused by different types of cancer. Among them, pancreatic ductal adenocarcinoma (PDAC) and cholangiocarcinoma are considered the two primary biliary stenosis malignancies. Approximately 70% of patients with PDAC show biliary obstruction^1^. The European Society of Gastrointestinal Endoscopy (ESGE) guidelines recommend routine preoperative biliary drainage in patients with MBO^2^. Biliary drainage to prevent cholangitis and severe jaundice is suggested as an adequate indication because a high level of bilirubin is associated with a high risk of postoperative complications^3^. The ESGE recommends that biliary drainage for DMBO be performed via endoscopic retrograde cholangiopancreatography (ERCP) rather than via surgery or percutaneously^2^, and stenting by ERCP is a standard practice of biliary drainage for DMBO^4^.

Recent ESGE guidelines strongly recommend the endoscopic placement of a 10-mm-diameter self-expandable metallic stent (SEMS) for preoperative biliary drainage of MBO because SEMSs are associated with a lower rate of reintervention than is the use of a plastic stent (PS) and based on a meta-analysis, there is no difference in overall surgery-related mortality or morbidity^2^. However, the interval between biliary drainage and surgery is not mentioned. Unlike the neoadjuvant chemotherapy setting, an upfront surgery strategy does not require a long wait time until surgery.

The Japanese guidelines for the management of biliary tract cancers recommend that preoperative biliary drainage is necessary for patients with jaundice and that endoscopic drainage is the most appropriate procedure due to the low risk of complications^5^. Nonetheless, these guidelines do not specify a stent type or size^5^. Haapamaki et al. reported that PSs do not differ from SEMSs with regard to stent dysfunction, decrease in bilirubin, or postoperative complications in a preoperative setting^6^.

To improve PS patency, a double-layer stent (DLS) was developed by Olympus Medical, and it is believed to be physically more patent than is a conventional PS. Therefore, we hypothesized that the patency of the 10-Fr DLS would be longer than that of a conventional PS with a smaller diameter and thus might be an alternative bridge to surgery (BTS) strategy for DMBO in an upfront surgery setting because this stent can maintain patency during the shorter duration of BTS. This study aimed to evaluate the efficacy and safety of a double layer, large-diameter PS in a BTS setting compared with a conventional small-diameter PS.

## Materials and methods

### Patients and strategy for preoperative biliary drainage

This was a single-center retrospective study performed between January 2011 and March 2018. Patients with extrahepatic DMBO scheduled to undergo definitive surgical treatment were included if they presented with increased serum bilirubin and transaminase levels or clinical symptoms due to obstructive jaundice. Biliary drainage was indicated for a case of acute cholangitis regardless of the degree of severity or of hyperbilirubinemia of 5 mg/dL or higher. However, if the patients were suspected to have bile duct cancer, they were indicated for biliary drainage for bile cytology irrespective of bilirubin level. Patients not meeting the above indications of biliary drainage were excluded. In addition, we excluded external biliary drainage including ENBD alone, who received SEMS for biliary drainage, or who were ultimately diagnosed with begin disease.

We commonly perform endoscopic placement of a nasobiliary drainage tube or a PS through ERCP as preoperative biliary drainage according to recommended guidelines^5^. The first endoscopic biliary drainage method involved a nasobiliary drainage tube or a PS depending on the clinical disease status of the patient or the physician’s preference. Regardless, a nasobiliary drainage tube was preferred when cholangiocarcinoma was suspected because of repeated cytology via a drainage tube, and a PS was preferred when PDAC was suspected because of another choice of tissue acquisition by endoscopic ultrasonography fine-needle aspiration. The endoscopic biliary drainage methods were performed using standard procedures with or without endoscopic sphincterotomy. Because our institution is a Japan Gastroenterological Endoscopy Society (JGES)-certified teaching hospital (No. 1239), trainees or experts performed ERCP to control cholangitis or jaundice caused by suspected MBO; trainees were assisted by experts as needed to avoid complications and to ensure procedural quality when performing ERCP using a duodenoscope (JF260 V or TJF-260 V: Olympus Optical Co. Tokyo, Japan). As a quality indicator for ERCP, we, including trainees at our hospital, have reported a post-ERCP pancreatitis rate of 3.9% (95% confidence interval 3.02–5.07%)^7^. We did not use SEMSs for preoperative biliary drainage of MBO.

Several types of PSs were employed, including a nasobiliary drainage tube at the initial ERCP for preoperative biliary drainage. After judging whether a patient was suitable for curative surgery, they received a PS if they had previously received a nasobiliary drainage tube. When biliary cannulation failed until surgery, we performed external biliary drainage.

During the study period, a total of 129 consecutive patients were newly diagnosed with histologically proven DMBO and underwent pancreaticoduodenectomy (PD) at Toyonaka Municipal Hospital.

The present study was conducted in accordance with the Declaration of Helsinki, and approval was obtained from the Institutional Review Board of Toyonaka Municipal Hospital (2017-10-06). This was a retrospective study involving personal data that were previously collected and did not require additional recruitment of human subjects; thus, the need for informed consent was waived via the *opt*-*out* method on our hospital’s website.

### PS types for preoperative biliary drainage

We divided the patients into two groups based on stent diameter: a double-layer, large-diameter plastic stent (LPS) group and a conventional small-diameter plastic stent (SPS) group. The LPS group received a 10-Fr DLS (Double-Layer stent, Olympus Medical Systems, Tokyo, Japan). The SPS group received a PS sized 7-Fr or 8.5-Fr in diameter (Flexima biliary stent, Boston Scientific Corporation, Boston, MA, USA, or Zimmon Biliary Stent, Cook, respectively). The choice of stent type and length was decided upon by the attending physician and was based on the patient's clinical and disease site characteristics.

### Definitions

The stent patency period started at the initial ERCP, which involved a nasobiliary tube or a PS. Stent dysfunction was defined as a requirement of stent replacement due to cholangitis or cholestasis from the initial replacement of a PS during surgery. Reintervention was performed as soon as possible when patients developed cholangitis or prolonged hyperbilirubinemia after PS replacement. Stents causing complications were removed, and the cause of dysfunction was determined by examining the removed stents. We grouped three causes of dysfunction: migration, occlusion of a stent with debris and occlusion of a stent with cholangitis. Occlusion of a stent with debris was considered when there were no findings of acute cholangitis, including clinical symptoms or inflammatory responses and increased serum bilirubin, transaminase levels or the presence of bile duct dilatation. If there were findings of cholangitis, the case was classified as occlusion of a stent with cholangitis. When stent dysfunction occurred, the use of a nasobiliary tube or a PS was allowed based on the physician’s judgment. The stent patency period was calculated as the interval between the initial nasobiliary tube or PS placement and its obstruction or operation. Total medical costs included all medical procedures, devices, and hospitalization, including any events of stent dysfunction until surgery.

### Endpoint and total cost evaluation and short-term outcomes of pancreaticoduodenectomy for DMBO

The primary endpoints in this study were the stent patency period and the stent patency rate at the median wait time until surgery. The secondary endpoints were complications related to stent placement, and clinical factors affecting stent dysfunction were explored. We also evaluated the short-term outcomes of pancreaticoduodenectomy for DMBO, including intraoperative data, postoperative complications, and mortality.

In addition, we assessed the clinical impacts of cost balance associated with preoperative biliary drainage for patients with DMBO between the LPS and SPS groups. The total medical cost evaluation from the initial diagnosis until just before surgery included costs for the total initial hospitalization (all stent placements, including secondary procedures) and secondary stent failure-related hospitalization until surgery, but it did not include costs related to the surgery and hospitalization. The total medical cost was calculated in Japanese yen (JPY) based on the database of the diagnosis procedure combination‐based payment system in Japan. One United States dollar (USD) was converted to 110.20 JPN (January 16, 2020).

### Statistical analysis

Continuous variables are reported as medians and interquartile ranges (IQRs). Categorical variables are summarized as frequencies (percentages). Differences in variables were evaluated using Fisher’s exact test for categorical data, and the Mann–Whitney U test was used to compare continuous variables. Patency was analyzed with the Kaplan–Meier method and the log-rank test, and differences between the LPS and SPS groups were examined. Risk factors for stent dysfunction were assessed using univariate and multivariate Cox proportional hazard models, and hazard ratios (HRs) and their 95% confidence intervals (CIs) are provided. All reported P values are two-sided, and P < 0.05 was considered significant. The statistical analyses were performed with JMP statistical software (ver. 14.3, SAS Institute, Inc., Cary, NC, USA).

## Results

### Patients

**Figure 1:**
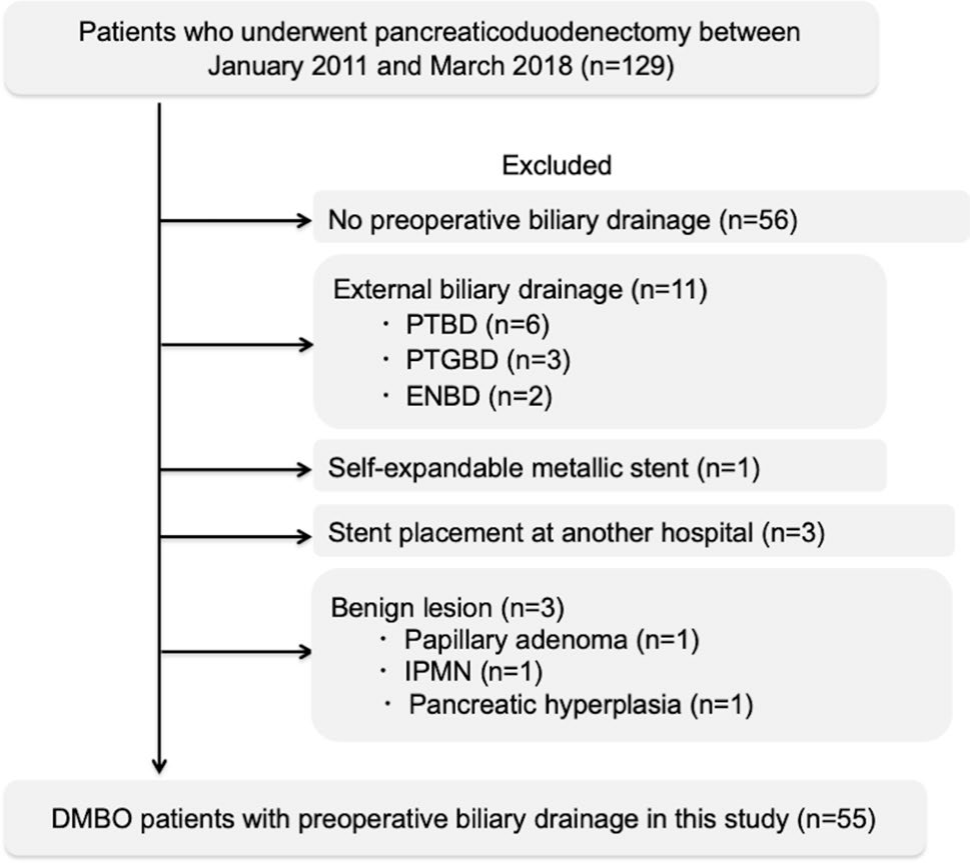
Flow chart of the patient selection. *PTBD* percutaneous transhepatic biliary drainage, *PTGBD* percutaneous transhepatic gallbladder drainage, *ENBD* endoscopic nasobiliary drainage, *IPMN* intraductal papillary mucinous neoplasm, *DMBO* distal malignant biliary obstruction.

Figure 1 presents the study flow chart. A total of 129 patients with DMBO were hospitalized at our hospital during the study period. Among them, 56 patients did not need preoperative biliary drainage (43.4%), 11 underwent external biliary drainage (8.5%) because of the difficulty of ERCP, one received an SEMS (0.8%), 3 received a PS at other hospitals (2.3%), and 3 were postoperatively diagnosed with benign lesions (2.3%). A nasobiliary drainage tube or PS when biliary cannulation was planned was successfully placed in all patients. We excluded 74 patients; thus, 55 patients (42.6%) who underwent PD and preoperative plastic biliary drainage for DMBO were enrolled and analyzed in the current study (Fig. 1).

The characteristics of all 55 patients with DMBO are provided in Table 1. The median age was 71 years, and 58% of patients were males. Sixteen patients (29%) had diabetes. Baseline diseases were bile duct carcinoma (N = 31), PDAC (N = 15), gallbladder cancer (N = 1), and papillary cancer (N = 8).

**Table 1:** Baseline characteristics of the patients enrolled in this study.

Variable	Total (n = 55)	LPS group (n = 19)	SPS group (n = 36)	P value
Age, median (IQR)	71 (63, 75)	72 (52–82)	70 (53–82)	0.4564
Sex, male, n (%)	32 (58)	9 (47)	23 (64)	0.2644
PS score, 0/1/2	42/12/1	16/3/0	27/8/1	0.6313
Diabetes mellitus, n (%)	16 (9.1)	8 (42.1)	8 (22.2)	0.2108
Body mass index, median (IQR)	21.6 (19.7, 23.6)	21.3 (19.4, 23.9)	21.8 (19.9, 23.6)	0.4155
**The cause of DMBO, n (%)**				
Bile duct carcinoma	31 (56.3)	9 (47.4)	22 (61.1)	0.5031
PDAC	15 (27.3)	6 (31.6)	9 (25)	
Gallbladder carcinoma	1 (1.8%)	1 (5.3)	0 (0)	
Papillary carcinoma	8 (14.5)	3 (15.8)	5 (13.9)	
**Final stage I/II/III/IV**	1/48/4/2	0/17/1/1	1/31/3/1	0.8215
Alb (g/dL), median (IQR)	3.6 (3.3, 3.9)	3.8 (3.3, 4)	3.6 (3.1, 3.8)	0.3332
T-Bil (mg/dL), median (IQR)	7.0 (3.3, 12.0)	6.5 (1.9, 9.1)	7.1 (3.8, 14.2)	0.2923
D-Bil (mg/dL), median (IQR)	4.9 (2.0, 9.8)	4.9 (0.60, 8.4)	5.6 (3.2, 11.5)	0.3040
AST (U/L), median (IQR)	148 (99, 290)	225 (99, 290)	144 (91, 293)	0.4360
ALT (U/L), median (IQR)	274 (130,430)	338 (151,430)	246 (118, 401)	0.2106
γGTP (U/L), median (IQR)	830 (420, 1538)	644 (424, 1,087)	1,051 (8,420, 1697)	0.4429
ALP (U/L), median (IQR)	1,337 (929, 1863	1,182 (883, 1722)	1,496 (637, 1,830)	0.3834
CRP (mg/dL), median (IQR)	0.50 (0.24, 1.87)	0.51 (0.15, 1.91)	0.55 (0.32, 1.87)	0.5880
FPG (mg/dL), median (IQR)	122 (103, 158)	125 (103, 219)	123 (100, 153)	0.3266
HbA1c (%) (NGSP), median (IQR)	5.9 (5.5, 6.5)	6.1 (5.8, 8.8)	5.8 (5.4, 6.2)	0.0693
CEA (ng/mL), median (IQR)	2.8 (1.8, 4.6)	2.6 (1.7, 6.3)	3.2 (2.2, 4.4)	0.6493
CA19-9 (U/mL), median (IQR)	95 (17, 359)	50 (16, 377)	146 (23, 411)	0.4525
The first endoscopic biliary drainage: ENBD, n, (%)	40 (72.7)	14 (74.7)	26 (72.2)	0.9077

Performance status was evaluated based on the Eastern Cooperative Oncology Group criteria.

*LPS* large plastic stent, *SPS* small plastic stent, *PS* performance status, *DMBO* distal malignant biliary obstruction, *PDAC* pancreatic ductal adenocarcinoma, *Alb* albumin, *T-Bil* total bilirubin, *D-Bil* direct bilirubin, *AST* aspartate aminotransferase, *ALT* alanine aminotransferase, *γGTP* γ-glutamyl transpeptidase, *ALP* alkaline phosphatase, *CRP* C-reactive protein, *FPG* fasting plasma glucose, *HbA1c* hemoglobin A1c, *CEA* carcinoembryonic antigen, *CA19-9* carbohydrate antigen 19-9, *ENBD* endoscopic nasobiliary drainage.

Among these patients, 36 and 19 received a small PS (SPS group) and a large PS (LPS group), respectively. There were no differences between the two groups (Table 1).

### Stent placement complications and stent dysfunction

Regarding the complications related to stent placement, four patients experienced mild pancreatitis: 3 patients (8%) received an SPS, and one patient received an LPS (9%). However, according to the Japanese guidelines for the management of acute pancreatitis, no severe ERCP-related pancreatitis developed^8^.

**Figure 2:**
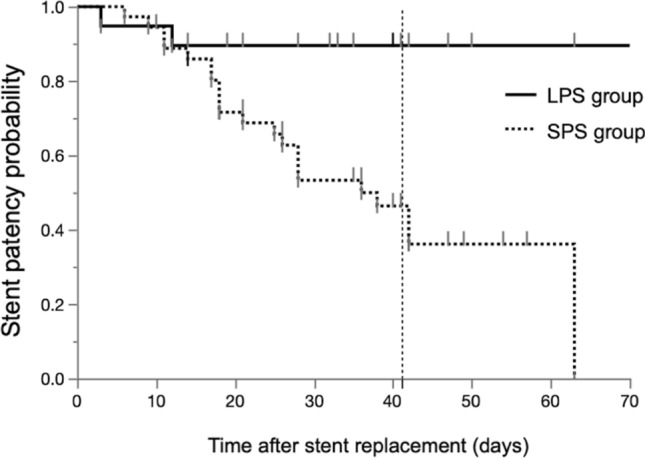
Stent patency probability between the large plastic stent and small plastic stent groups. The median wait time until surgery was 41 days. *LPS* large plastic stent, *SPS* small plastic stent.

The median interval time to surgery after stent placement was 41 days. There was no significantly different median interval from stent placement to surgery between the groups (LPS group 36 days vs. SPS group 41, P = 0.2494). No patients were lost during the follow-up period until surgery. Stent dysfunction before surgery occurred in a total of 23 patients. The patency rate of the LPS group was significantly higher than that of the SPS group (89.5% vs. 41.7%, P = 0.0006). The median number of stent dysfunction events was 0 in the LPS group and 1 in the SPS group, for a significantly lower rate in the LPS group than in the SPS group (P = 0.0034). The Kaplan–Meier curve also showed that stent patency was significantly longer in the LPS group than in the SPS group. Specifically, the 41-day stent patency rate (as stated, 41 days was the median interval time to surgery at our institution) was higher in the LPS group than in the SPS group (89% vs. 46%, P = 0.0034) (Fig. 2). The cause of stent dysfunction in the LPS group included migration of the stent (one patient) and occlusion of the stent with jaundice (one patient); the case with migration involved impaction at the cystic duct. The causes of stent dysfunction in the SPS group were occlusion of the stent with jaundice (two patients) and cholangitis (19 patients) (Table 2).

**Table 2:** Complications and stent dysfunction of plastic stent placement.

Variable	Total (n = 55)	LPS group (n = 19)	SPS group (n = 36)	P value
**Complication**				
Post-ERCP pancreatitis	4, 7.7%	1, 5.3%	3, 9.1%	1.000
**Stent dysfunction**				
Stent dysfunction, present, n (%)	23 (41.8)	2 (10.5)	21 (58.3)	0.0006
Median number of events, (IQR), range	0 (0,1), 0–3	0 (0,0), 0–3	1 (0.1), 0–3	0.0034
**Details of initial stent dysfunction, n (%)**				
Migration, n (%)	1 (1.8)	1 (5.3)	0 (0)	0.3455
Occlusion of a stent with debris, n (%)	3 (5.5)	1 (4.3)	2 (5.6)	1.0000
Occlusion of a stent with cholangitis, n (%)	19 (34.5)	0 (0)	19 (25)	< 0.0001
**Final stage I/II/III/IV**	1/48/4/2	0/17/1/1	1/31/3/1	0.8215
Interval to surgery, median (IQR)	41 (32, 42)	36 (28, 45)	41 (34, 49)	0.2530

*LPS* large plastic stent, *SPS* small plastic stent.

### Stent patency factors

We evaluated whether differences in clinical factors affected stent patency among patients with DMBO who received a PS for BTS. Univariate analysis showed that LPS use was significantly associated with longer stent patency [HR = 0.1524, 95% confidential interval (CI) 0.004–0.666, P = 0.0115] (Table 3). Multivariate analysis included stent group and diabetes with borderline significance related to stent patency adjusted for age and sex, revealing that LPS use was significantly associated with sufficient stent patency until surgery (HR = 0.103, 95% CI 0.022–0.470, P = 0.0033) (Table 3).

**Table 3:** Stent dysfunction for the preoperative interval in patients with DMBO based on univariate analysis and multivariate analysis adjusted by age.

	Univariate analysis			Multivariate analysis		
	HR	95% CI	P value	HR	95% CI	P value
Age	1.034	0.978–1.097	0.2435	1.014	0.965–1.072	0.5928
**Sex**						
Male	1			1		
Female	1.523	0.671–3.456	0.3142	1.478	0.568–3.849	0.4237
**PS score**						
0	1					
1/2	1.346	0.553–3.277	0.5125			
**Diabetes mellitus**						
None	1			1		
Present	2.080	0.906–4.775	0.0840	2.399	0.927–6.203	0.0711
BMI	1.023	0.903–1.162	0.7265			
**Primary cancer**						
Non-PDAC	1					
PDAC	1.233	0.483–3.150	0.6613			
**Stent group**						
SPS	1			1		
LSP	0.1524	0.004–0.666	0.0115	0.103	0.022–0.470	0.0033

*PDAC* pancreatic ductal adenocarcinoma, *LPS* large plastic stent, *SPS* small plastic stent.

### Short-term outcomes of pancreaticoduodenectomy for DMBO

No surgery-related death occurred among the cases. Additionally, there was no difference in the median reduction ratio of total bilirubin (presurgery levels/predrainage levels × 100) (15% in the LPS group and 12% in the SPS group, P = 0.2249). Table 4 shows the short-term outcomes of pancreaticoduodenectomy. Regarding intra- and postoperative data, there were no significant differences in operating time, blood loss, or postoperative complications between the SPS and LPS groups (Table 4).

**Table 4:** Intraoperative and postoperative data.

Variables	Total (n = 55)	LPS group (n = 19)	SPS group (n = 36)	P value
**Intraoperative data**				
Operation time (min), median (IQR)	433 (379, 501)	449 (388, 501)	430 (357, 502)	0.3482
Blood loss (mL), median (IQR)	665 (440, 950)	455 (340, 840)	670 (505, 976)	0.1214
**Postoperative data**				
Postoperative complication, present, n (%)	27 (49.0)	7 (37.8)	20 (55.6)	0.2588
All infection complications, n (%)	14 (25.5)	5 (26.3)	9 (25.0)	1.000
Surgical site infection, n (%)	7 (12.7)	2 (10.5)	5 (13.9)	1.000
Cholangitis, n (%)	3 (5.5)	1 (5.3)	2 (5.6)	1.000
Pancreatic fistula, n (%)	12 (21.8)	3 (15.8)	9 (25.0)	0.5114
Delayed gastric emptying, n (%)	6 (10.9)	2 (10.5)	4 (11.1)	1.0000
Postoperative mortality, n (%)	0 (0)	0 (0)	0 (0)	N.C

*LPS* large plastic stent, *SPS* small plastic stent, *N.C*: not calculated.

### Total medical costs until surgery

**Figure 3:**
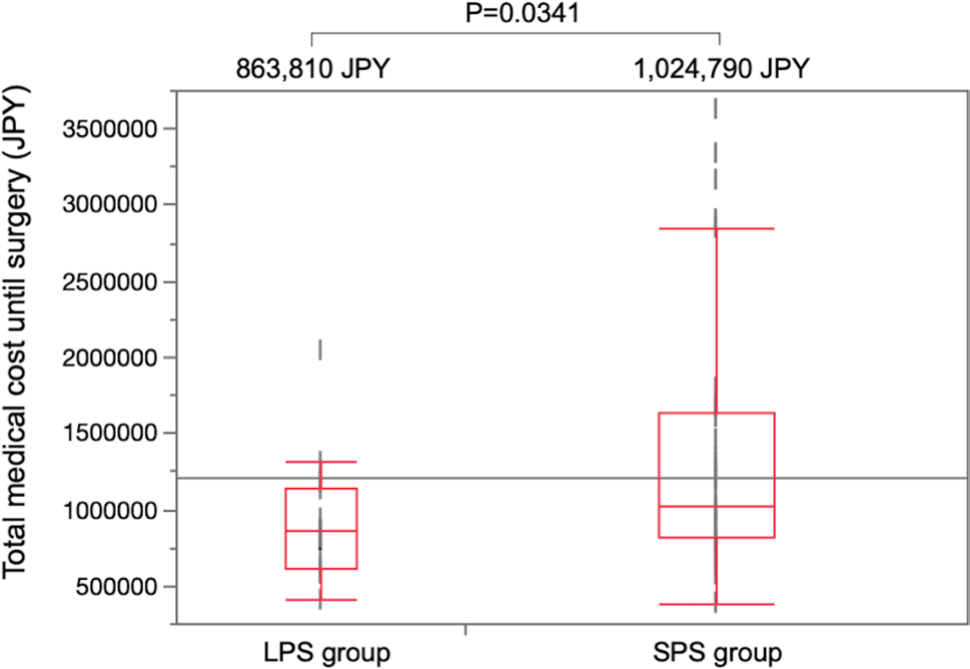
Total medical costs associated with preoperative biliary drainage until surgery were calculated based on the database of the diagnosis procedure combination‐based payment system in Japan. *LPS* large plastic stent, *SPS* small plastic stent, *JPY* Japanese yen.

As illustrated in Fig. 3, the total cost of LPS use was significantly lower than that of SPS use (863,810 JPY and 1,024,790 JPY, P = 0.0341).

## Discussion

We found that double-layer large-diameter PSs have significantly longer patency than do conventional small-sized PSs. A 10-Fr DLS may be used as an alternative for biliary drainage in the setting of upfront surgery for DMBO because it can maintain stent patency for at least 1 month prior to surgery. In contrast, the use of conventional smaller-sized PSs resulted in higher stent dysfunction and thus cannot be recommended. In approximately 90% of patients with DMBO who received 10-Fr DLSs, no reintervention until surgery was necessary. This was because the incidence of stent dysfunction was significantly lower in the LPS group than in the SPS group, despite no differences in terms of delay of surgery, decrease in bilirubin levels, procedures, or complications related to surgery. We believe that this lower incidence of stent dysfunction resulted in lower medical costs in the LPS group. Based on our results, instead of an SEMS, placement of a 10-Fr DLS can be considered an alternative method for preoperative biliary drainage of MBO.

The cost of SEMSs has been a topic of debate, and it is noted that the higher costs associated with the placement of SEMSs can be offset by a reduction in the costs of secondary procedures in cases of initial stent failure. A recent study demonstrated that the cost of stent strategies for managing DMBO depends on the survival of patients who are not candidates for resection. The use of a PS was less costly for patients with a survival time of less than 4 months, whereas the use of an SEMS was relatively less expensive for patients with prolonged survival^9^. In the future, we must compare the use of LPSs with SEMSs for patients with DMBO-planned PD based on medical costs.

A DLS has been developed to address the shortcomings of PSs, and it may be superior to a conventional PS in terms of patency ^10^. The DLS stent is composed of different material in each layer: the inner layer has a water repellent property that minimizes bile adhesion, and the double-layer design eliminates the flap and side holes to prevent bile accumulation in the stent lumen and four flaps at both the distal and duodenal ends to prevent stent migration. Isayama et al. showed that this stent could achieve relatively longer patency (approximately 4 months) in patients with nonresectable PDAC^11^. In a palliative setting, a meta-analysis of five studies that included 460 patients and three randomized control trials found longer stent patency but slightly more adverse events for DLS compared to conventional PS^3^.

Preoperative biliary drainage for MBO can reduce morbidity and mortality after surgery^12–15^. It is believed that obstructive jaundice due to MBO is related to the impairment of hepatic function, disturbances in coagulation, and the development of cholangitis^16^. However, two randomized trials and a systematic review showed that the overall complication rate in patients undergoing preoperative biliary drainage was higher than that in patients who directly underwent surgery^17,18^. This difference was partially explained by complications associated with the preoperative biliary drainage procedure itself.

Regarding which type of stent to choose, it has been reported that nearly 40% of PSs need stent exchange during the preoperative interval but that SEMSs require less endoscopic reintervention^4^. Randomized controlled trials have shown that SEMSs are superior to PSs for recurrent biliary obstruction^19,20^, and a meta-analysis revealed SEMSs to be associated with a lower rate of endoscopic reintervention compared with PSs (3.4% vs. 14.8%), though with no difference in overall surgical morbidity or mortality^21^. Moreover, Walter et al. reported that the total cost of PS use was similar to that of SEMS use in patients with a short survival duration (≤ 3 months) or in those with metastatic disease^22^. Therefore, the ESGE strongly recommends the placement of a 10-mm-diameter SEMS for preoperative biliary drainage of MBO^2^. The Japanese guideline for the management of biliary tract cancers in 2014 states that endoscopic drainage is the most appropriate procedure but does not comment on which type of stent should be used, though endoscopic nasobiliary drainage (ENBD) is recommended for biliary drainage in patients with hilar/proximal bile duct carcinoma who are scheduled to undergo extended hepatectomy^5^. Therefore, Japanese endoscopists tend to prefer to use a PS for preoperative biliary drainage because of the lower cost.

The present study has several limitations due to its retrospective nature. First, the number of subjects was small, and the stent type was chosen by the attending physician based on the patient's clinical and disease site characteristics. Second, the attending physician in this study included experts and trainees; however, all ERCPs were conducted under supervision by expert endoscopists. Third, this study did not compare the 10-Fr DLS with the 10-mm-diameter SEMS. Finally, differences in patient populations (cholangiocarcinoma is likely not as common in Western countries) and practice patterns (European guidelines are based on studies in which 50% of patients are treated with neoadjuvant chemotherapy) prevent the generalization of our findings to other groups. The median time to surgery in this cohort was 41 days; thus, neoadjuvant therapy was not common.

This single-center retrospective study showed that a double-layer, large PS has significantly longer patency than a small PS and can maintain stent patency during the wait time until upfront surgery for DMBO.
